# Reduced Fluoresceinamine as a Fluorescent Sensor for Nitric Oxide

**DOI:** 10.3390/s100301661

**Published:** 2010-03-02

**Authors:** Abel J. Duarte, Joaquim C.G. Esteves da Silva

**Affiliations:** 1 REQUIMTE, Instituto Superior de Engenharia do Porto, R. António Bernardino Almeida 431, 4200-072 Porto, Portugal; E-Mail: ajd@isep.ipp.pt; 2 Centro de Investigação em Química, Departamento de Química e Bioquímica, Faculdade de Ciências da Universidade do Porto, Rua Campo Alegre 687, 4169-007 Porto, Portugal

**Keywords:** reduced fluoresceinamine, NO sensor, fluorescence, fiber optics

## Abstract

A new fluorescent sensor for nitric oxide (NO) is presented that is based on its reaction with a non fluorescent substance, reduced fluoresceinamine, producing the highly fluorescent fluoresceinamine. Using a portable homemade stabilized light source consisting of 450 nm LED and fiber optics to guide the light, the sensor responds linearly within seconds in the NO concentration range between about 10–750 μM with a limit of detection (LOD) of about 1 μM. The system generated precise intensity readings, with a relative standard deviation of less than 1%. The suitability of the sensor was assessed by monitoring the NO generated by either the nitrous acid decomposition reaction or from a NO-releasing compound. Using relatively high incubation times, the sensor also responds quantitatively to hydrogen peroxide and potassium superoxide, however, using transient signal measurements results in no interfering species.

## 1. Introduction

The determination of reactive oxygen and nitrogen species (RONS) is the object of study of numerous physiological and pathological processes [1,2]. The production of RONS, which includes nitric oxide (NO), is mediated by multiple oxidative stress mechanisms responsible for changes in the cells and tissues [3–6]. Beside this role in these pathological processes, RONS compounds are involved in numerous physiological process of regulation in the organism [7,8]. Elevated steady state concentrations of these species, on the other hand, can lead to the disruption of these cellular mechanisms. NO is well-known as a vasodilator and as an inhibitor of platelet activation and aggregation [9]. In this context, the development of advanced analytical tools with the ability to detect NO in real time, with high sensitivity, specificity and reliability is critical for better understanding of their impact in the human health.

Several analytical methodologies, for example chemiluminescence, colorimetry, electrochemical, fluorescence and electron spin resonance, have been proposed for *in vitro* and *in vivo* analysis of NO [10–13]. The potential main advantage of molecular fluorescence-based methods is its intrinsic sensitivity coupled to designed sensor selectivity and relatively low cost of the instrumentation necessary for analysis. Moreover, molecular fluorescence is compatible to fiber optics instrumental designs, which open interesting perspectives for *in vivo* measurements. One promising fluorescent sensor for NO is based on diaminofluorescein (DAF) [10–13], although selectivity problems have been raised regarding the application of this sensor for *in vivo* measurements [10,11]. 2-[6-(4′-hydroxy)phenoxy-3*H*-xanthen-3-on-9-yl]benzoic acid and 2-[6-(4′-amino)phenoxy-3*H*-xanthen-3-on-9-yl]benzoic acid have been proposed as novel fluorescent probes to detect selectively highly reactive ROS [14]. Reduced 1′,7′-dichlorodihydrofluorescein (DCFH_2_) and dihydrorhodamine 123 (DHR) are often used to detect the production of oxidizing species in cells *via* oxidation to their fluorescent products [15,16]. Also, reduced fluorescein has been used for the determination of hydrogen peroxide [17].

**Figure 1.**
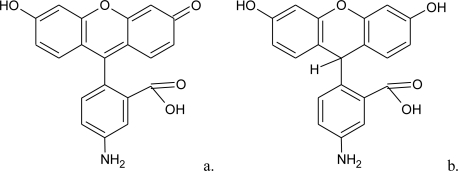
Chemical structures of fluoresceinamine (a) and probable reduced fluoresceinamine (b).

Fluoresceinamine (Figure 1a) is a common and relatively inexpensive fluorescence indicator that can be easily immobilized in solid supports [18–20]. During research for its immobilization and reactivity when immobilized, it was observed that, when in solution, fluoresceinamine is easily reduced by solid zinc in acid medium [21], producing a non-fluorescent substance. Also, it was observed that only few compounds reacted with reduced fluoresceinamine. Some of these substances belong to the RONS family of compounds, including NO. Based on the reduction of DCFH_2_ and DHR [15], a probable structure for reduced fluoresceinamine is shown in Figure 1b.

**Figure 2.**
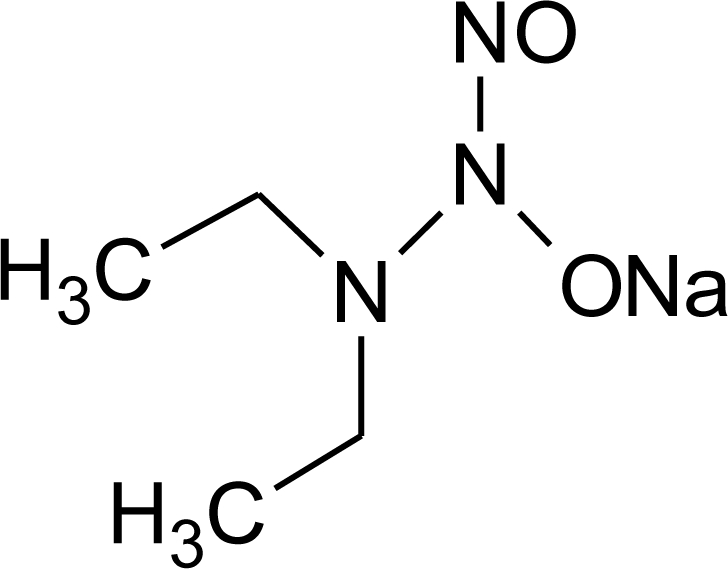
Chemical structure of DEANO.

The objective of this paper is to describe a simple and inexpensive method for NO detection and quantification based on the fluorescence development resulting from the reaction of NO with non-fluorescent reduced fluoresceinamine. This system is tested and validated using NO generated either by nitrous acid decomposition reaction or by hydrolysis of diethylamine NONOate (DEANO, Figure 2) in aqueous solution.

## 2. Results and Discussion

Preparation of the reduced fluoresceinamine aqueous solution (sensor) is straightforward since it is the direct product of the reaction between fluoresceinamine and solid zinc in acid medium, and the reduction can be visually monitored because the unreduced chemical form is bright yellow and the reduced form has no color. The sensor solution is readily obtained after centrifugation and dilution in buffer solution.

**Figure 3.**
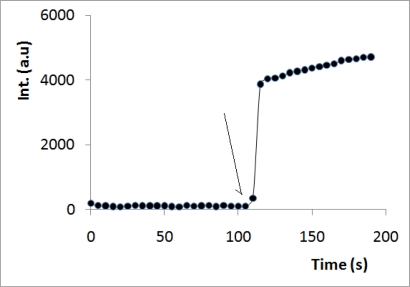
Fluorescence profile (excitation at 450 nm and emission at 522 nm) before and after mixing NO with reduced fluoresceinamine (the arrow shows the point were NO was added).

Figure 3 shows the time course of the fluorescence intensity before and after the addition of NO-containing solution (obtained from dissolving an NO-releasing compound (DEANO) in phosphate buffer solution and incubation for one hour at 37 °C) to the sensor. Figure 3 shows that the system responds to NO in less than 10 seconds (reaction and solution homogenizing), which is quite a fast response for a fluorescent sensor.

In order to assess the analytical potential of the sensor, a portable homemade stabilized light source constituted by 450 nm LED and fiber optics (1 mm diameter glass) to guide the light coupled to a commercial charge-coupled device detector was used (USB4000 OceanOptics). Two detection methods were tested (full details in the experimental section): one method with an incubation time of one hour (sensor plus NO-containing solution) before fluorescence measurement; and, another method with the sensor inside a fluorescent quartz cell in front of the detector (acquiring data) and the NO-containing solution added to it. It was observed that the reaction between the sensor and NO was faster in the presence of a cobalt(II) ion that acts as a catalyst (for the same reaction time the fluorescence intensity more than doubled), consequently, this ion was always added.

### 2.1. Sensor Response to NO

**Figure 4.**
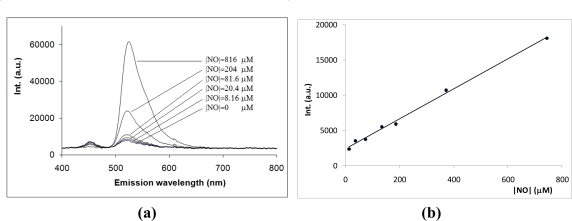
(a) Fluorescence emission spectra of reduced fluoresceinamine in the presence of increasing amounts of NO (excitation 450 nm); and, (b) typical calibration plot for NO (excitation at 450 nm and emission at 522 nm).

In order to assess if the NO sensor responds quantitatively, several solutions were prepared using different DEANO solutions. Figure 4a shows the emission spectra resulting from the reaction of reduced fluoresceinamine and increasing NO concentrations after one hour reaction time. As shown, the fluorescence intensity increased with the concentration of NO. Also, the fluorescence emission spectra of the product of the reaction between reduced fluoresceinamine and NO is similar to that of fluoresceinamine (maximum at 522 nm), which suggests that NO oxidizes the sensor.

Figure 4b shows a typical calibration curve of the fluorescence intensity after one hour reaction time at 37 °C as a function of the concentration of NO. A detailed analysis of this figure shows the existence of a linear relationship in the concentration range between about 10–750 μM with a limit of detection (LOD) of about 1 μM (correlation coefficient higher than 0.998). Similar characteristics were observed when the NO-containing solution was added to the sensor solution in the spectrophotometric cell and the fluorescence intensity was measured in the following minutes after homogenization, although using this method measurements were not as reproducible.

To study the reproducibility of the system under development, series of different NO solutions were analyzed (380, 94 and 38 μM.) The analysis of the maximum fluorescence for the three sets show a relative standard deviation of the fluorescent signal of about 1%, 0.7% and 0.5% for the higher, average and lower concentration, respectively. These results show that the system generates results with quite good precision.

### 2.2. Application of the Sensor to NO Measurements

**Figure 5.**
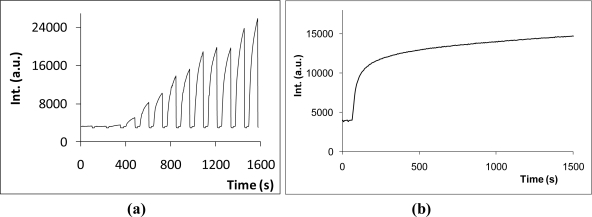
Detection NO present in aqueous solution as a result of production from the reaction of nitrite and sulfuric acid (a) and from the hydrolysis of DEANO (b).

In order to assess the suitability of the sensor to monitor NO in aqueous solutions, NO gas generation by two systems were evaluated: (i) NO in an aqueous solution resulting from the reaction of nitrite and sulfuric acid (nitrous acid decomposition) (Figure 5a); and (ii) from the hydrolysis of DEANO (Figure 5b).

Figure 5a shows a series of measurements of NO sample solutions taken from a reactor where NO is being bubbled. As expected, the concentration of NO in solution is increasing (the intensity at 522 nm is increasing) with the time. This result shows the suitability of the sensor to monitor NO in aqueous solution. Figure 5b shows the increase of the fluorescence intensity when a freshly prepared DEANO solution was added to the sensor solution. The first order kinetic analysis of the intensity as function of the time allows the estimation of a half-life of 12±1 min^−1^, which is similar to the literature value of 16 min^−1^ [22]. This result confirms and validates that the reduced fluoresceinamine is a sensor for NO detection.

### 2.3. Analysis of Interfering Species

**Figure 6.**
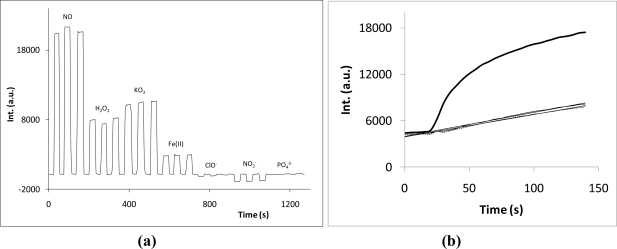
Interfering species assessment (concentration of all species is about 400 μM): (a) steady state fluorescence after one hour; and (b) initial fluorescence variation of the sensor in contact with NO (▬), H_2_O_2_ (^…^) and KO_2_ (─).

To evaluate the effect of possible chemical interferences in the fluorescent signal, 400 μM solutions of NO, hydrogen peroxide, potassium superoxide, iron(II), sodium hypochlorite, sodium nitrite and phosphate (used as blank) were prepared and the response of the sensor solution to these solutions analyzed. The background fluorescence was subtracted from all the fluorescent signals. As shown in Figure 6a, hydrogen peroxide (about 38% of the NO signal) and potassium superoxide (about 49% of the NO signal) developed marked fluorescence after one hour in contact with the sensor. Iron(II) also showed a significant effect (about 10% the NO signal). The other species provoked fluorescent variation smaller than 5% of the NO signal.

However, this interference study was done with the sensor and candidate interfering species in contact for one hour at 37 °C. As discussed previously, the sensor response to NO is quite fast. As shown in Figure 6b the reaction between the sensor and the two most interfering species (hydrogen peroxide or potassium superoxide) was much slower than that observed for NO under similar concentrations. Indeed, in the first few minutes no marked fluorescence increase is detected. This property can be quite useful in developing discrimination strategies to make the sensor highly selective for NO. Two straightforward analytical solutions can be proposed: (i) reducing the incubation time of the sensor and the sample under analysis to some minutes; or, (ii) performing transient signal measurement using a flow injection analysis technique.

## 3. Experimental Section

Fluoresceinamine, Diethylamine NONOate sodium salt hydrate (DEANO), zinc powder, trishydroxylmethylammonium chloride, trisodium citrate and potassium superoxide were purchased from Sigma-Aldrich Química S. A. (Spain). Cobalt(II) chloride standard solution, hydrogen peroxide 30% solution, potassium dihydrogen orthophosphate, sodium phosphate dibasic, sodium nitrite, sodium hydroxide, sodium hypochlorite 4% solution and hydrogen chloride were obtained from Merck (Germany). Deionized water was used in all experiments. Tris pH = 7.4 buffer was made by 50 mM trishydroxylmethylammonium chloride and 20 mM trisodium citrate with the pH adjusted with sodium hydroxide. Phosphate pH = 7.4 buffer was made by 322 mM potassium dihydrogen orthophosphate and 6.8 mM sodium phosphate dibasic with the pH adjusted with sodium hydroxide. With the exception of the fluoresceinamine solution, all the solutions were prepared in phosphate buffer and prepared daily. Fluoresceinamine solution was prepared by weighing followed by dissolution in 0.10 M hydrogen chloride.

The chemical reduction of fluoresceinamine followed the following procedure: (i) 1.4 mL of a solution of 1.45 mM fluoresceinamine and 14.0 mL of 0.10 M hydrogen chloride were mixed in a 15 mL Falcon tube; (ii) 0.5 g of zinc powderwas added; (iii) the Falcon tube was stirred until the fluorescence and the yellow color disappeared. After centrifugation, the obtained reduced fluoresceinamine solution was diluted (5.0 mL) with 10 mL of Tris buffer solution (pH = 7.4) and 4.7 mL 0.10 M sodium hydroxide. This sensor solution is stable for at least two hours.

Two NO gas generation methods were used: (i) a concentrated standard solution of DEANO was prepared by dissolving 7 mg of solid DEANO in deoxygenated (bubbling nitrogen for at least 1 hour) phosphate buffer up to 10.00 mL—successive dilutions were prepared in phosphate buffer. (ii) A saturated aqueous solution of sodium nitrite was prepared by weighing 22 g of the salt, placing it in a close vessel, then adding 6 M sulfuric acid dropwise to the saturated nitrite solution from a burette attached to the vessel. The gas that is generated is washed with a 20% sodium hydroxide solution and finally bubbled in the phosphate buffer. Prior to addition of the sulfuric acid solution the system is deoxygenated by bubbling nitrogen for at least one hour.

Two detection methods of NO solution with reduced fluoresceinamine were tested: (i) 2.40 mL of the diluted reduced fluoresceinamine solution was transferred to the standard fluorescence quartz cell followed successively by 0.10 mL CoCl_2_ and 0.10 mL of a NO-containing solution—the solution inside the cell in constant stirring; (ii) 4.8 mL of the diluted reduced fluoresceinamine was transferred to a 20 mL vial and stirred continuously in a thermostatic bath at 37 °C. After 30 min, 0.20 mL of 0.01131 M cobalt(II) chloride solution and 0.20 mL of a DEANO solution were added. This solution was left stabilizing for about one hour before the fluorescence was measured. The fluorescence measurement was made in standard quartz fluorescence cells with a magnetic stirring bar.

Fluorescence measurements were made with an homemade equipment containing a stabilized light source constituted of 450 nm LEDs from Roithner Lasertechnik (Ref. LED450-01); a CCD detector from Ocean Optics (USB4000); a sampling compartment from Ocean Optics (CUV-ALL-UV 4-way); a 1.0 mm glass fiber optic to guide the light from the source to the sampling compartment; a 0.600 mm core diameter fiber optic (P600-2-UV-VIS from OceanOptics) to guide the emitted light from the sampling compartment to the detector.

Linear regression calculations were done with Microsoft-Excel spreadsheet. LOD was calculated using the following criteria: LOD = (3s/b), where s is the standard deviation of 16 blank measurements and b is the slope of the calibration curve. For kinetic studies the rate of NO formation was determined by the intensity (*Int*.) increase of the fluorescent band at 522 nm and the rate constant (*k*) was obtained from the plots of ln(*Int*_∞_-*Int*) *vs. t*. A first order was observed using about 350 data points in the time interval between and 2,000 seconds with a correlation coefficient higher than 0.99. At least duplicated runs were obtained and the half-time calculated *t*_1/2_ = 0.6931/*k*.

## 4. Conclusions

A new fluorescence sensor for nitric oxide based on the reaction of reduced fluoresceinamine is proposed and assessed. The low cost, selectivity and rapid response characteristics of the sensor towards NO confer it great potential for the development of screening tests. Although using the instrumental configuration in this work allows quantitative NO concentration estimations down to the micromolar concentration range, the limits of detection can be decreased several orders of magnitude by using more sensitive equipment.

Also, the versatility of the sensor can allow further analytical developments, namely: fluoresceinamine can be easily immobilized on solid supports allowing the development of immobilized NO sensors; the immobilization of this sensor on the tip of a fiber optic would allow *in vivo* NO detection; and finally, flow analytical methodologies with improved analytical performance can be easily implemented using this sensor.
